# Causes Preventive of Maternal Nursing

**Published:** 1911-04-01

**Authors:** 


### Diseases of Children.

### CAUSES PREVENTIVE OF MATERNAL NURSING.

Three causes are mainly operative in depriving infants of their right to the breast, exclusive of such conditions as harelip or other similar abnormality in the child. First, there may be physical inability on the mother's part to nurse her child. Secondly, there may be inability on her part by reason of her engagement in some industrial pursuit. Thirdly, the mother may have disinclination, usually by reason of the trouble maternal nursing involves, and the separation from social pleasures and pursuits it entails.

Von Bunge has shown that, apart from local and systematic disease, alcoholism seems to be the chief cause in any country as a whole which renders mothers as a class unable to nurse their children. The daughters of the third generation of alcoholics are usually unable to suckle their young.

The engagement of the mother in some industrial pursuit depends partly upon the willingness of husbands to accept the earnings of their wives at the expense of their children. This forces the mother to work for her bread while her child has to be fed upon the bottle by someone else.

Much can be done by general popular enlightenment to eliminate the third cause—namely, the disinclination of mothers to nurse their children. It is hardly to be supposed that many women will refuse to nurse their babies from purely selfish considerations, once they are fully informed as to the enormous advantages breast feeding confers upon a child. It is obviously the duty of the medical profession to further this by every means at their command. Every mother should nurse her child unless there are very cogent reasons to the contrary.

The following causes may be mentioned as contra-indicating maternal nursing: (1) When no milk is secreted; (2) tuberculosis, latent or active—a tuberculous mother who suckles her child not only tends to accelerate the progress of her own disease, but also exposes the child to the danger of contracting it; (3) when the mother is affected by grave systematic disease; (4) when the mother is choleric or epileptic; (5) when the mother has suffered from a severe complication of the parturient state, such as eclampsia nephritis, extreme hæmorrhage, puerperal septicæmia, and the like; (6) local disease of the mammary gland; (7) when as the result of two previous experiences under favourable conditions she has shown her inability to nurse her child.

